# Associations between Adolescents’ Interpersonal Relationships, School Well-being, and Academic Achievement during Educational Transitions

**DOI:** 10.1007/s10964-019-01184-y

**Published:** 2019-12-31

**Authors:** Noona Kiuru, Ming-Te Wang, Katariina Salmela-Aro, Lasse Kannas, Timo Ahonen, Riikka Hirvonen

**Affiliations:** 1 Department of Psychology, University of Jyvaskyla, P.O. Box 35, 40014 Jyväskylä, Finland; 2 University of Pittsburg, Pittsburgh, PA USA; 3 Department of Educational Science, University of Helsinki, Helsinki, Finland; 4 Faculty of Sport and Health Sciences, University of Jyväskylä, Jyväskylä, Finland; 5 School of Applied Educational Science and Teacher Education, University of Eastern Finland, Joensuu, Finland

**Keywords:** Early adolescence, School well-being, Interpersonal relationships, Academic achievement, Educational transition

## Abstract

A youth’s ability to adapt during educational transitions has long-term, positive impacts on their academic achievement and mental health. Although supportive relationships with parents, peers, and teachers are protective factors associated with successful educational transitions, little is known about the reciprocal link between the quality of these interpersonal relationships and school well-being, with even less known about how these two constructs affect academic achievement. This longitudinal study examined how the quality of interpersonal relationships and school well-being worked together to affect academic achievement during the transition from primary school to lower secondary school. Data were collected from 848 Finnish adolescents (54% girls, mean age at the outset 12.3 years) over the course of sixth and seventh grade. The results support a transactional model illustrating the reciprocal associations between the quality of interpersonal relationships and school well-being during the transition to lower secondary school. As such, the presence of high quality interpersonal relationships promoted higher academic achievement through increased school well-being, whereas high school well-being promoted higher subsequent academic achievement through increased quality of interpersonal relationships. Overall, the results suggest that promoting learning outcomes and helping adolescents with challenges during educational transitions is a critical part of supporting school well-being and the formation of high-quality interpersonal relationships.

## Introduction

Educational transitions from primary to lower secondary school pose potential risks for declining learning motivation and academic achievement (Eccles 2004). Understanding the mechanisms that protect adolescents from disengagement during critical educational transitions is important, because successful adaptation to the new educational context predicts the completion of higher education, better job prospects, and higher life satisfaction (for a review see Upadyaya and Salmela-Aro 2013). The stage–environment fit model suggests that a poor fit between changes in individual (e.g., an intensified need for autonomy; a heightened need for social acceptance and support when facing changes related to puberty) and contextual (e.g., stricter grading practices and increasingly distant relationships with teachers) levels may hinder adolescents’ adaptation during educational transitions (Eccles 2004; Hill and Wang 2015). Supportive relationships with parents, school friends, and teachers constitute one possible protective factor that may facilitate successful educational transitions (e.g., Burchinal et al. 2008; Waters et al. 2014) because these positive relationships can promote students’ feelings of relatedness and facilitate adaptation to a new school environment (Ryan and Deci 2017). Although some theoretical suggestions have been posited on how interpersonal relationships, school well-being, and academic achievement may be related (Sameroff 2009), little is known about the reciprocal dynamics between the quality of interpersonal relationships and school well-being, and how these dynamics contribute to academic achievement. A better understanding of how quality of interpersonal relationships and school well-being work together to affect academic achievement during educational transitions is crucial, since successful adaptation during the critical transitions has long-term impacts on youth’s academic and mental-health outcomes (Upaydyaya and Salmela-Aro 2013). Consequently, this study examined (a) the longitudinal associations between quality of interpersonal relationships and school well-being, and (b) the mediating mechanisms through which quality of interpersonal relationships and school well-being combine to predict students’ subsequent academic achievement during the transition to lower secondary school.

### Adolescent School Well-being and Interpersonal Relationships

School is a central developmental context in early adolescents’ lives. In fact, school can be seen as adolescents’ main workplace, characterized by similar features to those of adults, such as standard tasks and activities, deadlines, work responsibility, and feedback routines (Samdal 2017). Similar to the adult workplace, adolescents contend with experiences that cause anxiety or stress, hence affecting their well-being. School well-being plays a significant role not only in current and future well-being and health conditions (Bond et al. 2007; Samdal et al. 2004) but also in subsequent educational outcomes (Upadyaya and Salmela-Aro 2013). In this study, adolescent school well-being is defined as school satisfaction and stress that are thought to reflect key aspects of adolescents’ emotional experiences of the school environment. School satisfaction describes the overall positivity of adolescents’ school experiences and refers to the liking, enjoyment, and interest associated with school (Eccles 2004; Huebner and Gilman 2006); school stress is defined as students’ experiences of school-related expectations and demands that exceed their inner resources and endanger their well-being (Salmela-Aro and Upadyaya 2014; Sonmark and Modin 2017).

School well-being is inextricably linked to the school’s social context (Sameroff 2009). The most prominent interpersonal relationships during the adolescent years involve friends and teachers at school and parents at home (Eccles and Roeser 2011; Moore et al. 2018; Wentzel 1998). Interpersonal support may be paramount during the transition from primary school to lower secondary school, not least because this change coincides with multiple challenges and changes in the organization and social structure of the educational setting (Eccles 2004). Although adolescents start to establish independence from parents while concurrently investing more time and energy into peer relationships (Steinberg and Morris 2001), parents still serve as important role models, as adolescents build their identities and adjust to new roles and responsibilities (Castro et al. 2015). While peer and teacher relationships are subject to change during this transitional period, parental support tends to remain stable (see also Hill and Wang 2015). Nevertheless, school friends and teachers form primary sources of social support in the school context. Teachers can be seen as temporary attachment figures serving as a safe haven and a secure basis for students (Verschueren 2015). However, particularly peers make students’ time at school enjoyable (Kindermann 2016) and adolescence is a developmental period characterized by the heightened desire to “fit in” with peers (Hamm and Zhang 2010).

School-based relationships with teachers and peers have been shown to influence school well-being, and this influence is stronger than that exacted by more distal macro-level factors, such as income and social-background related factors (Ottová-Jordan et al. 2015; Park et al. 2012). Adolescents themselves have also identified social interactions within the school community as simultaneously being the most rewarding yet the most challenging part of their school careers (Pyhältö et al. 2010). Despite possible changes in these relationships during transitional periods, maintaining supportive relationships with school friends and teachers may play a pivotal role in adolescent school well-being and successful adaptation to a new educational context (see also Longobardi et al. 2016; West et al. 2010).

Adolescents not only form relationships with teachers and peers, they also enter into a dynamic, reciprocal relationship with their school environment. The transactional model (Sameroff 2009) suggests that adolescent development is a product of the continuous dynamic interactions between adolescents and the experiences provided by their social settings (e.g., parents, friends, teachers). The core of the transactional model lies in the interdependent effects of the adolescent and the environment, which are depicted in the reciprocal associations between the adolescent and others. On the one hand, the extent of perceived social support (e.g., closeness and conflict) from parents, school friends, and teachers may promote or undermine adolescent school well-being (i.e., socialization effects). On the other hand, adolescent school well-being may also elicit reactions from significant others, thereby influencing the quality of interpersonal relationships (see also Kerr and Stattin 2003; Nurmi and Kiuru 2015).

The associations between adolescents’ school-based interpersonal relationships and school well-being have been well established in the literature (e.g., Baker et al. 2003; Quin 2017; Wang and Eccles 2012). However, the extant research is mostly limited to studies that use cross-sectional designs or examine only unilateral relationships (i.e., either the effect of interpersonal relationships on school well-being—socialization effects—or the effect of school well-being on interpersonal relationships—evocative effects), thus making it difficult to draw conclusions about the direction of associations. Hence, less is known about the reciprocal associations between adolescent school well-being and their perceived quality of interpersonal relationships, particularly during the transition from primary to lower secondary school. Along the same line, only a few studies have simultaneously investigated adolescents’ interpersonal relationships with different social agents (i.e., parents, friends, teachers) and the effects of both positive (e.g., closeness) and negative (e.g., conflict) aspects of these relationships. According to the attachment-based relationship model (see Pianta 2001; Verschueren 2015) closeness and conflict are considered key dimensions of relationship quality. Closeness is characterized by high levels of warmth and trustworthiness between adolescents and their significant others, whereas conflict refers to strained and conflictual interactions with a negative tone within the relationship.

### Interpersonal Relationships, School Well-being, and Academic Achievement

Supportive interpersonal relationships function as an important resource for promoting students’ academic skill development. According to the self-determination theory (SDT), supportive relationships may fulfill the adolescent’s basic psychological need for social relatedness (Deci and Ryan 2000). When this need is met, adolescents feel connected to their teacher and peers, which fosters their motivation to behave in socially appropriate ways and concentrate on learning. Interpersonal support may also reduce stress in demanding situations and increase adolescent’s focus on and interest in learning tasks (Kiuru et al. 2014; Wang and Eccles 2012). The transactional dynamics between the quality of interpersonal relationships and school well-being may also impact adolescents’ academic performance, though no prior studies have investigated these mechanisms during the critical transition from primary to lower secondary school.

Among the studies targeting different age groups, it has been shown that children’s classroom engagement is an important mediator between their feelings of relatedness to peers, parents, and teachers and their academic achievement (Wang et al. 2019). In another study, supportive interpersonal environments with peers, parents, and teachers were shown to promote primary school children’s academic achievement through increased task-focused behavior (Kiuru et al. 2014). Similarly, the effects of adolescent perceptions of supportive relationships with peers and teachers and the sense of school belonging have been shown to predict later academic achievement via academic engagement (Zimmer-Gembeck et al. 2006). Although rarely examined, the experiences of school well-being might also impact the quality of interpersonal relationships (see also Nurmi and Kiuru 2015), which in turn may have consequences for adolescents’ subsequent academic achievement. To the authors’ best knowledge, no previous cross-lagged longitudinal studies have investigated whether perceived quality of relationships with parents, friends, and teachers together with school well-being predict adolescents’ academic achievement during school transitions. A better understanding of these dynamics could provide researchers, educators, and policy-makers means for preventing negative academic development that tend to co-occur during this critical transition.

## Current Study

**Fig. 1:**
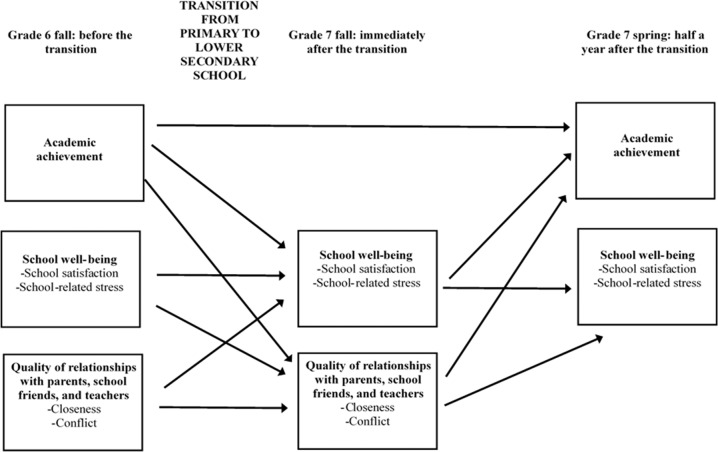
Schematic model for the role of associations between quality of interpersonal relationships, school well-being, and academic achievement.

To overcome the limitations of previous research, this study aimed to investigate transactional associations between interpersonal relationships, school well-being, and academic achievement during the critical transition from primary to lower secondary school (for schematic figure, see Fig. 1). The first research question was to examine the reciprocal relationship between adolescents’ perceived quality (i.e., closeness, conflict) of their relationships with parents, school friends, and teachers, and school well-being (i.e., school satisfaction, school stress). It was expected that (a) high levels of closeness to and low levels of conflict with parents, school friends, and teachers would predict increased school well-being, and that (b) high school well-being would predict increased closeness to and decreased conflict with parents, friends, and teachers. The second research question was to examine the extent to which the quality of relationships with parents, school friends, and teachers predicted adolescents’ academic achievement through school well-being. It was hypothesized that high closeness to and low conflict with parents, friends, and teachers would predict improved academic achievement through increased school well-being. The final research question was to examine the extent to which school well-being predicted adolescents’ academic achievement through the perceived quality of their interpersonal relationships. It was hypothesized that high school well-being would predict improved academic achievement through increased closeness to and decreased conflict with parents, school friends, and teachers.

## Method

### Participants and Procedure

This study analyzed data from a broader longitudinal study that follows a community sample of Finnish students in Central Finland across the transition from primary to lower secondary school. Finnish children start their education at kindergarten during the year of their sixth birthday. One year later, at age 7, they move to comprehensive school where they continue for the next 9 years. Comprehensive school divides into a lower level (grades 1–6) and an upper level (grades 7–9). In the Finnish school system, the transition from primary (grades 1–6) to lower secondary school (grades 7–9) marks the first remarkable transition for students. The transition to lower secondary school marks a change in the school environment including multiple changes, such as increased workload, often shifting to other school buildings, and always moving from a classroom teacher system to a subject teacher system with increased amount of new teachers and classmates. For the present study, primary schools were selected from areas where all the children transfer to particular secondary schools instead of dispersing to different locations (see also Mauno et al. 2018).

The sample of this study consisted of 848 adolescents (457 girls, 54%) who were examined both before (i.e., grade 6) and after (i.e., grade 7) their transition to lower secondary school. These adolescents came from 56 school classes, ranging in size between seven and thirty pupils (*M* = 21.1, SD = 4.66). A total of 91% of adolescents (*n* = 770) filled in the questionnaires at all the three time points (*n* = 827 in the fall of the sixth grade; *n* = 802 in the fall of the seventh grade; *n* = 793 in the spring of the seventh grade); 9% of adolescents (*n* = 78) completed the questionnaires only once or twice out of three time points.

At the beginning of the study, the participants were in the fall semester of the sixth grade, and their ages ranged from 11 to 13 (*M=* 12.3 years, SD = 4.36 months). The participants’ mother tongue was Finnish in 98% of the cases. The sample was fairly representative of the Finnish general population in regard to demographic characteristics (Official Statistics of Finland 2016a, 2016b).

Data were collected during normal school days in the 2014–2016 academic years. Information on adolescent school well-being and perceived quality of interpersonal relationships was collected using questionnaires. All the questionnaires were administered by trained testers with two trained research assistants being present at all the test situations. Information about the participants’ academic achievement (i.e., school grades) was obtained from the school registers. The study has been evaluated and approved by the ethics committee of the University of Jyväskylä (February 12, 2014).

### Measures

#### School Well-being (Grade 6 Fall; Grade 7 Fall; Grade 7 Spring)

The adolescents reported their school-related well-being using the items adapted from the Health Behavior in School-aged Children (HBSC) Study (Currie et al. 2012; see also Kämppi et al. 2012). School satisfaction was assessed using three items (e.g., “I enjoy going to school”; *α* = 0.88–0.90), and school stress was measured using four items (e.g., “I have too much schoolwork”; *α* = 0.78–0.81) on a 5-point Likert scale (*1* = *completely disagree*; *5* = *completely agree*).

#### Closeness to and Conflict with Parents (Grade 6 Fall; Grade 7 Fall)

The adolescents were asked to rate their experienced closeness (five items; e.g., “I have a close and warm relationship with my mother/father”) and conflict (six items; e.g., “I often argue with my mother/father”) with their mothers and fathers using the Child Parent Relationship Scale (CPRS; Driscoll and Pianta 2011; see also Mauno et al. 2018). The adolescents answered the questions on a five-point Likert scale (*1* = *not true at all*; *5* = *completely true*). The mean scores were calculated to measure the adolescents’ perceived closeness to and conflict with their mothers (*α* = 0.83–0.86; *α* = 0.79–0.86) and fathers (*α* = 0.86–0.87; *α* = 0.77–0.86), respectively.

#### Closeness to and Conflict with School Friends (Grade 6 Fall; Grade 7 Fall)

The adolescents were asked to rate their experiences of closeness (seven items; e.g., “I feel happy when spending time with my friend”; *α* = 0.86–0.88) and conflict (four items; e.g., “My friend and I argue a lot”; *α* = 0.75–0.79) with their best friends at school using the Friendship Qualities Scale (Bukowski et al. 1994). The adolescents answered these questions using a five-point Likert scale (*1* = *not true at all*; *5* = *completely true*).

#### Closeness to and Conflict with Teachers (Grade 6 Fall; Grade 7 Fall)

The adolescents were asked to rate their closeness (five items; e.g., “I have a close and warm relationship with my teacher”; *α* = 0.80–0.82) and conflict (six items; e.g., “I often argue with my teacher”; *α* = 0.77–0.84) with their sixth-grade classroom teacher during the 2014 fall semester and with their seventh-grade literacy, math, and other teachers during the 2015 fall semester using the Student-Teacher Relationship Scale (STRS-Short Form; Pianta 2001; see also Jerome et al. 2008). The adolescents answered the questions on a five-point Likert scale (*1* = *not true at all*; *5* = *completely true*). The mean scores were calculated across these ratings to attain an estimate of the adolescents’ overall perceptions of their closeness to and conflict with their teachers.

#### Academic Achievement (Grade 6 Fall; Grade 7 Spring)

Information on the students’ grade point average was acquired from the school registers in the fall of the sixth grade and spring of the seventh grade. In Finnish schools, the possible grades range from 4 to 10, with 5 being the lowest passing grade and 10 the highest passing grade.

### Statistical Analyses

The descriptive statistics were analyzed first (see Table 1). Then the measurement models were estimated by using confirmatory factor analysis separately for each variable, measuring school well-being (i.e., school satisfaction, school stress) and perceived quality of interpersonal relationships (i.e., closeness to and conflict with parents, school friends, and teachers). The measurement models of school well-being consisted of three time points (i.e., Grade 6 fall, Grade 7 fall, and Grade 7 spring), and the measurement models of the quality of interpersonal relationships consisted of two time points (i.e., Grade 6 fall and Grade 7 fall). In these measurement models, factor loadings of the same items were constrained to be equal across time to ensure invariance of the measurement across time, and the latent factors were allowed to correlate with each other. Next, the cross-lagged structural equation models for school well-being, perceived quality of interpersonal relationships, and academic achievement were estimated. In these models, factor loadings of the same items were constrained to be equal across time to ensure invariance of the measurement across time. Finally, the direct and indirect effects were estimated with the full cross-lagged structural equation models for school well-being, quality of interpersonal relationships, and academic achievement.

**Table 1:** Means and standard deviations of the study variables.

	*n*	*M*	*SD*	Range of scale
School satisfaction (gr6, fall)	835	3.39	0.90	1–5
School satisfaction (gr7, fall)	799	3.65	0.88	1–5
School satisfaction (gr7, spring)	781	3.34	0.94	1–5
School-related stress (gr6, fall)	835	2.50	0.81	1–5
School-related stress (gr7, fall)	799	2.46	0.84	1–5
School-related stress (gr7, fall)	781	2.77	0.86	1–5
Closeness to parents (gr6, fall)	838	3.95	0.83	1–5
Closeness to parents (gr7, fall)	801	3.74	0.92	1–5
Closeness to friends (gr6, fall)	833	4.13	0.72	1–5
Closeness to friends (gr7, fall)	790	4.17	0.71	1–5
Closeness to teachers (gr6, fall)	835	2.30	0.83	1–5
Closeness to teachers (gr7, fall)	792	2.25	0.75	1–5
Conflict with parents (gr6, fall)	839	2.04	0.73	1–5
Conflict with parents (gr7, fall)	801	1.88	0.78	1–5
Conflict with friends (gr6, fall)	833	1.91	0.74	1–5
Conflict with friends (gr7, fall)	788	1.85	0.75	1–5
Conflict with teachers (gr6, fall)	837	1.63	0.68	1–5
Conflict with teachers (gr7, fall)	792	1.40	0.56	1–5
Academic achievement (gr6, fall)	694	8.25	0.66	5–10
Academic achievement (gr7, spring)	768	8.14	0.89	5–10

*gr6* 6th grade, *gr7* 7th grade

The statistical analyses were performed using the Mplus statistical package (Version 8; Muthén and Muthén 1998–2018) with the COMPLEX approach to account for the clustered nature of the data (Muthén and Muthén 1998–2018; see also Asparouhov and Muthén 2006; Muthén and Satorra 1995). For the indirect effects, the coefficient estimates, standard errors, and p values were reported using the COMPLEX approach. In addition, a bootstrapping procedure was used to confirm the indirect effects and their 95% confidence intervals (MacKinnon et al. 2004).

The proportion of missing data for the main study variables ranged from 2% to 18.2% (*M* = 5.26; SD = 3.91). The parameters of the models were estimated using full-information maximum likelihood (FIML) with non-normality robust standard errors (maximum likelihood robust, MLR; Muthén and Muthén 1998–2018). The goodness-of-fit of the estimated models was evaluated using the following absolute goodness-of-fit indices: (a) *χ*^*2*^ test, (b) root mean square error of approximation (RMSEA), (c) standardized root mean square residual (SRMR), and (d) comparative fit index (CFI). The acceptable fit was defined as RMSEA ≤ 0.08 and CFI ≥ 0.90 (Browne and Cudeck 1993; Hoyle 1995; Hu and Bentler 1999).

## Results

### Measurement Models

The measurement models were built using confirmatory factor analysis, enabling to take measurement error into account in the primary analyses. These models were built separately for school satisfaction, school-related stress, and for perceived closeness and conflict in relationships with parents, friends, and teachers. In these models, factor loadings of the same items were constrained to be equal across time to ensure invariance of the measurement across time. If required for model fit, some autocovariances of residuals of the same items were estimated. The measurement models, assuming measurement invariance across time, fit the data well: *χ*^2^(1–73) = 0.67–258.80–59.62, *ps=* 0.00–0.79, RMSEAs = 0.00–0.06, CFIs = 0.94–1.00, and SRMRs = 0.03–0.06. The standardized estimates of factor loadings for the key constructs were high (i.e., no factor loading was lower than 0.40). The fact that the models fit the data well with high factor loadings suggests good structural validity and item reliability.

### Structural Equation Models for Interpersonal Relationships and School Well-being

The first aim of this research was to investigate the extent to which the adolescents’ perceived quality of interpersonal relationships predict their subsequent school well-being and vice versa. Table 2 presents the correlations between the latent factors in Grades 6 and 7 and academic achievement. As the aim was to get a distinct picture of the negative and positive aspects of the perceived interpersonal relationships, the cross-lagged SEM models for school well-being were carried out separately for relationship closeness and conflict variables.

**Table 2:** Correlations of latent factors of school well-being and perceived relationship quality with academic achievement.

	1	2	3	4	5	6	7	8	9	10	11	12	13	14	15	16	17	18	19	20
1. School satis-faction (gr6, fall)	–																			
2. School satis-faction (gr7, fall)	0.66^a^	–																		
3. School satis-faction (gr7, spring)	0.60^a^	0.67^a^	–																	
4. School stress (gr6, fall)	−0.65^a^	−0.49^a^	−0.41^a^	–																
5. School stress (gr7, fall)	−0.52^a^	−0.63^a^	−0.38^a^	0.62^a^	–															
6. School stress (gr7, spring)	−0.45^a^	−0.43^a^	−0.54^a^	0.53^a^	0.72^a^	–														
7. Closeness to parents (gr6, fall)	0.34^a^	0.33^a^	0.26^a^	−0.21^a^	−0.26^a^	−0.22^a^	–													
8. Closeness to parents (gr7, fall)	0.28^a^	0.33^a^	0.33^a^	−0.23^a^	−0.32^a^	−0.31^a^	0.59^a^	–												
9. Closeness to friends (gr6, fall)	0.35^a^	0.34^a^	0.21^a^	−0.17^a^	−0.15^a^	−0.08	0.44^a^	0.37^a^	–											
10. Closeness to friends (gr7, falll)	0.31^a^	0.40^a^	0.24^a^	−0.18^a^	−0.24^a^	−0.20^a^	0.36^a^	0.48^a^	0.67^a^	–										
11. Closeness to teachers (gr6, fall)	0.39^a^	0.26^a^	0.22^a^	−0.24^a^	−0.20^a^	−0.15^b^	0.26^a^	0.41^a^	0.19^a^	0.30^a^	–									
12. Closeness to teachers (gr7, fall)	0.28^a^	0.40^a^	0.34^a^	−0.14^c^	−0.33^a^	−0.26^a^	0.47^a^	0.35^a^	0.31^a^	0.19^a^	0.48^a^	–								
13. Conflict with parents (gr6, fall)	−0.28^a^	−0.24^a^	−0.20^a^	0.37^a^	0.31^a^	0.26^a^	−0.26^a^	−0.35^a^	−0.08^c^	−0.08^c^	−0.15^b^	−0.07	–							
14. Conflict with parents (gr7, fall)	−0.22^a^	−0.26^a^	−0.26^a^	0.34^a^	0.42^a^	0.25^a^	−0.25^a^	−0.48^a^	−0.14^b^	−0.20^a^	−0.21^a^	−0.13^c^	0.58^a^	–						
15. Conflict with friends (gr6, fall)	−0.17^a^	−0.15^b^	−0.08	0.28^a^	0.19^a^	0.19^a^	−0.10^c^	−0.13^c^	−0.14^a^	−0.15^a^	−0.05	−0.06	0.40^a^	0.36^a^	–					
16. Conflict with friends (gr7, fall)	−0.20^a^	−0.33^a^	−0.20^a^	0.32^a^	0.37^a^	0.32^a^	−0.23^a^	−0.24^a^	−0.19^a^	−0.28^a^	−0.15^a^	−0.09^c^	0.34^a^	0.48^a^	0.54^a^	–				
17. Conflict with teachers (gr6, fall)	−0.43^a^	−0.29^a^	−0.24^a^	0.40^a^	0.32^a^	0.25^a^	−0.18^a^	−0.22^a^	−0.18^a^	−0.22^a^	−0.23^a^	−0.10^c^	0.53^a^	0.35^a^	0.28^a^	0.24^a^	–			
18. Conflict with teachers (gr7, fall)	−0.37^a^	−0.44^a^	−0.32^a^	0.41^a^	0.50^a^	0.32^a^	−0.26^a^	−0.28^a^	−0.26^a^	−0.25^a^	−0.15^a^	−0.02	0.33^a^	0.54^a^	0.22^a^	0.42^a^	0.50^a^	–		
19. Ac. achievement (gr6, fall)	0.30^a^	0.32^a^	0.25^a^	−0.32^a^	−0.22^a^	−0.21^a^	0.13^b^	0.16^a^	0.25^a^	0.19^a^	0.05	0.05	−0.13^a^	−0.07	−0.17^a^	−0.18^a^	−0.34^a^	−0.24^a^	–	
20. Ac. achievement (gr7, spring)	−40^a^	0.40^a^	0.38^a^	−0.33^a^	−0.30^a^	−0.32^a^	0.18^a^	0.23^a^	0.29^a^	0.25^a^	0.10^c^−	0.11^c^	−0.16^a^	−0.15^a^	−0.14^a^	−0.20^a^	−0.41^a^	−0.28^a^	0.84^a^	–

*gr* grade

^a^*p* < 0.05; ^b^*p* < 0.01; ^c^*p* < 0.001

#### Closeness of Interpersonal Relationships and School Well-being

The final model for the adolescents’ perceived closeness of interpersonal relationships and school well-being fit the data well: *χ*^2^(1081) = 2008.64, *p<* 0.001, CFI = 0.94, RMSEA = 0.03, SRMR = 0.05. After controlling for the stabilities of the constructs, the results illustrated that the cross-lagged paths from closeness to parents (*β* = 0.07, SE = 0.03, *p* = 0.049) and to school friends (*β* = 0.10, SE = 0.04, *p* = 0.006) in the fall of the sixth grade to school satisfaction in the fall of the seventh grade, and from closeness to parents in the fall of the seventh grade (*β* = 0.11, SE = 0.04, *p* = 0.002) to school satisfaction in the spring of the seventh grade were positive. Furthermore, the cross-lagged paths from closeness to parents in the fall of the sixth grade (*β* = −0.12, SE = 0.05, *p* = 0.011) to school stress in the fall of the seventh grade, and from closeness to parents in the spring of the seventh grade (*β* = −0.09, SE = 0.04, *p* = 0.021) to school stress in the spring of the seventh grade were negative. Finally, cross-lagged paths from school satisfaction in the fall of the sixth grade to the subsequent perceived closeness to parents (*β* = 0.15, SE = 0.03, *p* < 0.001), friends (*β* = 0.10, SE = 0.04, *p* = 0.007), and teachers (*β* = 0.09, SE = 0.04, *p* = 0.033) in the fall of the seventh grade were positive. In addition, a significant indirect effect (estimate = 0.023, SE = 0.010, *p* = 0.024) was detected from high school satisfaction in the fall of the sixth grade to increased school satisfaction in the spring of the seventh grade through increased closeness to parents in the fall of the seventh grade. Similarly, an indirect effect (estimate = −0.014, SE = 0.006, *p* = 0.024) from high school satisfaction in the fall of the sixth grade to decreased school stress in the spring of the seventh grade through increased closeness to parents in the fall of the seventh grade was significant.

#### Conflict in Interpersonal Relationships and School Well-being

The final model for the adolescents’ perceived conflict in interpersonal relationships and school well-being fit the data well: *χ*^2^(909) = 1394.80, *p* < 0.001, CFI = 0.97, RMSEA = 0.03, SRMR = 0.05. The cross-lagged path from conflict with teachers in the fall of the sixth grade to school stress in the fall of the seventh grade (*β* = 0.08, SE = 0.04, *p* = 0.037) was positive, whereas the cross-lagged path from conflict with parents in the fall of the seventh grade to school satisfaction in the spring of the seventh grade (*β* = −0.07, SE = 0.03, *p* = 0.024) was negative. Similarly, the cross-lagged paths from school stress in the fall of the sixth grade to the subsequent perceived conflict with parents (*β* = 0.15, SE = 0.04, *p* < 0.001), friends (*β* = 0.19, SE = 0.03, *p* < 0.001) and teachers (*β* = 0.25, SE = 0.04, *p* < 0.001) in the fall of the seventh grade were positive. In addition, a significant indirect effect (estimate = 0.019, SE = 0.008, *p* = 0.017) was detected from low school satisfaction in the fall of the sixth grade to decreased school satisfaction in the spring of the seventh grade through increased conflict with parents in the fall of the seventh grade. Similarly, an indirect effect (estimate = −0.014, SE = 0.007, *p* = 0.047) from high school stress in the fall of the sixth grade to decreased school satisfaction in the spring of the seventh grade through increased conflict with parents in the fall of the seventh grade was significant.

### Structural Equation Models for Interpersonal Relationships, School Well-being, and Academic Achievement

As the final step, the participants’ academic achievement was incorporated into the previously estimated models for perceived quality of interpersonal relationships and school well-being.

#### Models for Closeness

**Fig. 2:**
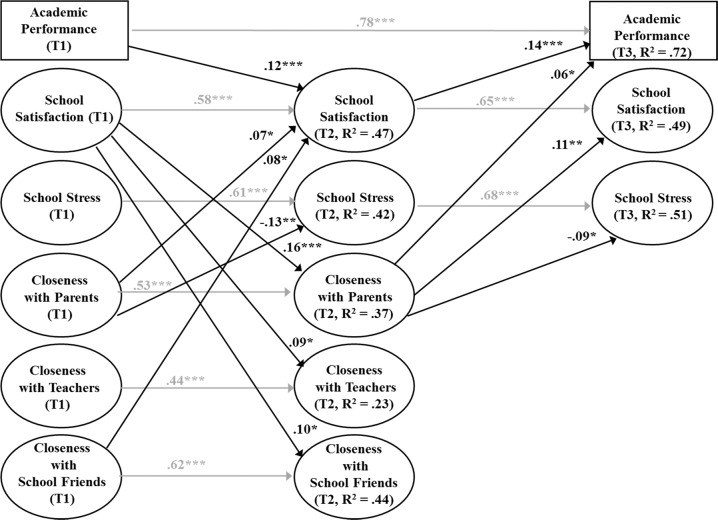
Final model of closeness of interpersonal relationships, school well-being, and academic achievement. *Note*. Fit of the model: *χ*^2^(1169) = 2232.82, *p* < 0.001, CFI = 0.95, RMSEA = 0.03, SRMR = 0.05. T1 = Grade 6 fall, T2 = Grade 7 fall, T3 = Grade 7 spring. Estimates are shown as standardized estimates. Constructs and residuals of the constructs within the same time points were allowed to correlate. *p < 0.05; **p < 0.01; ***p < 0.001.

The final combined SEMs for closeness in interpersonal relationships, school well-being, and academic achievement is shown in Fig. 2. The results for cross-lagged paths between closeness in interpersonal relationships and school well-being were same than those reported in the previous models (see Fig. 2). In addition, two significant cross-lagged effects for academic achievement were found. First, high academic achievement predicted the adolescents’ increased school satisfaction in the fall of the seventh grade. Second, high school satisfaction in the fall of the seventh grade predicted the adolescents’ increased academic achievement in the spring of the seventh grade. Three statistically significant indirect effects were detected as well (see Table 3): the adolescents’ high closeness to their parents and friends in the fall of the sixth grade indirectly promoted the subsequent academic achievement in the spring of the seventh grade through increased school satisfaction in the fall of the seventh grade. In addition, adolescents’ high school satisfaction in the fall of the sixth grade indirectly promoted the subsequent academic achievement in the spring of the seventh grade through increased closeness to parents in the fall of the seventh grade.

**Table 3:** Estimates of indirect effects on academic achievement: school well-being in school and quality of interpersonal relationships as mediators (*N* = 848).

Indirect effect	Estimate	SE
From quality of interpersonal relationships via well-being in school to academic achievement		
Closeness to parents (T1)→School satisfaction (T2)→Academic achievement (T3)	0.012*	0.006
Closeness to school friends (T1)→School satisfaction (T2)→Academic achievement (T3)	0.022*	0.011
From well-being in school via quality of interpersonal relationships to academic achievement		
School satisfaction (T1)→Closeness to parents (T2)→Academic achievement (T3)	0.009*	0.004
School stress (T1)→Conflict with teachers (T2)→Academic achievement (T3)	−0.027***	0.007

*p < 0.05; ***p < 0.001

#### Models for Conflict

**Fig. 3:**
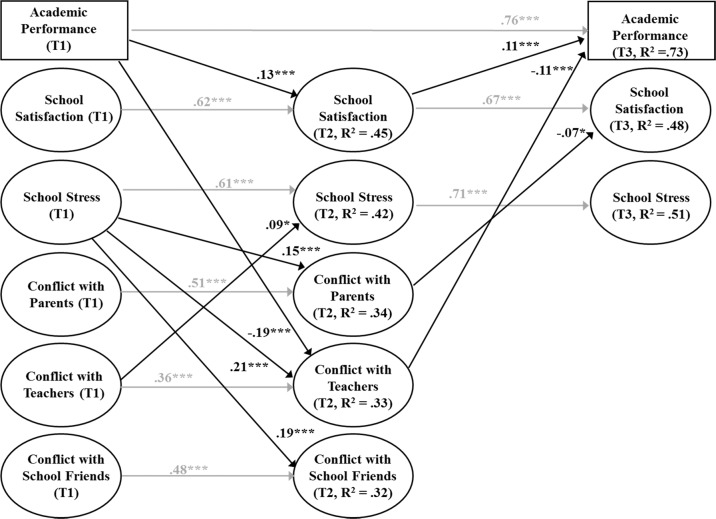
Final model of conflict in interpersonal relationships, school well-being, and academic achievement. *Note*. Fit of the model: *χ*^2^(988) = 1561.75, *p* < 0.001, CFI = 0.96, RMSEA = 0.03, SRMR = 0.05. T1 Grade 6 fall, T2 Grade 7 fall, T3 Grade 7 spring. Estimates are shown as standardized estimates. Constructs and residuals of the constructs within the same time points were allowed to correlate. *p < 0.05; **p < 0.01; ***p < 0.001.

The final combined SEMs for conflict in interpersonal relationships, school well-being, and academic achievement is shown in Fig. 3. The results for cross-lagged paths between conflict in interpersonal relationships and school well-being were same than those reported in the previous models (see Fig. 3). In addition, four significant cross-lagged effects for academic achievement were found. The first two effects were same than in the model for closeness: high academic achievement predicted the adolescents’ increased school satisfaction in the fall of the seventh grade and high school satisfaction in the fall of the seventh grade predicted the adolescents’ increased academic achievement in the spring of the seventh grade. In addition, low academic achievement predicted the adolescents’ increased conflict with teachers in the fall of the seventh grade. Finally, increased conflict with teachers in the fall of the seventh grade predicted the adolescents decreased academic achievement in the spring of the seventh grade. Also one statistically significant indirect effect was found (see Table 3): school stress in the fall of the sixth grade indirectly hindered subsequent academic achievement in the spring of the seventh grade through increased conflict with teachers immediately after their transition in the fall of the seventh grade.

### Additional Analyses

As additional analyses, the similar analyses reported above were also carried out separately for each type of relationship (school friends, parents, and teachers) without controlling for the effects of other types of relationships. The main pattern for the transactional associations between quality of interpersonal relationships and school well-being was similar as reported above. In addition, two additional paths were detected in the models of both relationship closeness and conflict that were not significant when controlling for relationship types with each other. Furthermore, one additional indirect effect was detected. These differences in the findings are explained in more detail in the following.

#### Models for Closeness

A significant cross-lagged effect from high closeness with teachers in the fall of sixth grade to subsequent higher school satisfaction in the fall of seventh grade was detected: (*β* = 0.08, SE = 0.04, *p* = 0.041). In addition, a significant cross-lagged effect from high closeness with friends on subsequent higher academic achievement in the spring of seventh grade was detected (*β* = 0.05, SE = 0.02, *p* = 0.034).

#### Models for Conflict

A significant cross-lagged effect from high conflict with parents in the fall of sixth grade and subsequent higher school stress in the fall of seventh grade was detected: (*β* = 0.08, SE = 0.04, *p* = 0.032). Moreover, a significant cross-lagged effect from high conflict with parents in the fall of seventh grade and subsequent lower academic achievement in the spring of seventh grade was detected: (*β* = −0.06, SE = 0.02, *p* = 0.003). In addition, the indirect effect (estimate = −0.01, SE = 0.004, *p* = 0.008) from high stress in the fall of sixth grade on subsequent lower academic achievement in the spring of seventh grade through increased conflict with parents was significant.

## Discussion

Despite the importance of educational transitions for adolescents’ academic and well-being outcomes (Wang and Eccles 2012), little is known about the transactional dynamics of interpersonal relationships, school well-being, and academic achievement during the critical transition to lower secondary school. This study examined these dynamics in the school context. The results revealed that closeness to parents and school friends promoted school well-being, while conflict with teachers hindered it. In addition, high levels of school satisfaction promoted relationships with parents, school friends, and teachers, while these relationships were hindered by high levels of school stress. The results also showed that adolescents’ high quality interpersonal relationships promoted their higher academic achievement through increased school well-being, whereas adolescents’ high school well-being promoted their higher academic achievement through increased quality of interpersonal relationships. The results provide novel understanding about reciprocal dynamics of quality of adolescent interpersonal relationships, school well-being and academic achievement during critical educational transition.

### Transactional Associations between Interpersonal Relationships and School Well-being during the School Transition

The results generally supported the authors’ hypotheses and the transactional model (Sameroff 2010). However, the pattern of results differed, depending on whether positive (i.e., closeness or school satisfaction) or negative (i.e., conflict or school stress) aspects of interpersonal relationships and school well-being were examined and in which phase of the transition the associations were observed. In essence, the results illustrated that supportive relationships with parents continued to play a remarkable role when the adolescents moved from primary to lower secondary school. The expected transactional associations were found between the adolescents’ relationships with their parents and school well-being. High perceived closeness to (but not perceived conflict) with parents predicted the adolescents’ higher subsequent school well-being (in regard to high school satisfaction and low school stress), whereas high school well-being predicted higher quality relationships with parents. The results suggested that support and encouragement from parents are helpful for adolescents when facing the challenging transition from primary to lower secondary school (see also Castro et al. 2015; Duineveld et al. 2017; Pina and Gonzales 2014). The relationships between the adolescents and their parents are not necessarily interrupted at transition to the same extent as that of the relationships with their school friends and teachers. Hence, parental support may be more consistently available during transition than support from changing school friends and teachers (Wang et al. 2013; Virtanen et al. 2019).

The expected transactional associations were also detected between the adolescents’ school well-being and relationships with school friends (Sameroff 2009): the adolescents’ perceived closeness to (but not conflict with) their school friends positively predicted their subsequent school satisfaction (but not school stress). Similarly, the adolescents’ high school well-being predicted a higher level of perceived closeness to and a lower level of conflict with school friends. These results suggest that closeness to school friends acts as a particularly promoting factor for the adolescents’ school satisfaction, thus highlighting the importance of peer relationships for the adolescents’ school well-being. Moreover, school well-being also strongly impacted adolescents’ perceived closeness to and conflict with school friends, possibly via transmission of positive and negative emotions (e.g., Aunola et al. 2015), which play a role in the quality of interactions between adolescents and their school friends.

Furthermore, the expected transactional associations were detected between the school well-being and relationships with teachers (Sameroff 2009). These results revealed that particularly a high level of conflict with teachers predicted the adolescents’ increased school stress, while closeness to teachers had no effect. This finding suggests that conflict with teachers is particularly detrimental to school well-being, while closeness does not play such an important role, perhaps because of the adolescents’ more distant relationships with teachers (Wang et al. 2013) perhaps partly due to discontinuity of teacher-student relationships across the transition (Virtanen et al. 2019). In addition, the adolescents’ high school well-being contributed to increased closeness to and decreased conflict with teachers.

### Transactional Associations of Interpersonal Relationships and School Well-being and Subsequent Academic Achievement

The final aim of this study was to investigate how transactional dynamics between interpersonal relationships and school well-being might predict the adolescents’ academic achievement during the critical transition to lower secondary school. In line with the expectations, the results indicated that high closeness to parents before the transition indirectly promoted the adolescents’ higher subsequent academic achievement through increased school satisfaction after the transition. One mechanism through which the pre-transition social support from parents may affect the students’ post-transition adaptation at school involves support continuity between the primary and lower secondary school environments. Social support is typically available from the family across the transition and, therefore, it may have longitudinal effects on promoting school well-being and academic outcomes (see Upadyaya and Salmela-Aro 2013; Wang et al. 2011). Parents may also influence adolescents’ positive school attitudes and academic achievement by modeling academically oriented behaviors, socializing an achievement orientation, and representing positive values regarding education (Anderson et al. 2007; Castro et al. 2015).

Congruent with the hypotheses, high closeness to friends before the transition indirectly promoted the adolescents’ higher subsequent academic achievement through increased school satisfaction after the transition. When maintained across the transition, close and supportive relationships with friends are a readily available source of continuity in the new school environment, which promotes students’ adaptation after the transition (Aikins et al. 2005; Kingery et al. 2011). It has been suggested that support from friends before the transition can act as a protective factor after the transition in at least two ways (Hirsch and Dubois 1992; see also Virtanen et al. 2019). First, adolescents with close and supportive relationships with school friends before the transition are more likely to maintain satisfactory post-transition friend networks, and these networks protect them from exposure to potential threats in a new school environment. Second, adolescents may draw upon their earlier sense of support from school friends, employing it as an emotional bank account when facing transition-related challenges.

A high level of conflict with teachers immediately after the transition was also found to directly undermine the adolescents’ subsequent academic achievement after the transition, whereas the adolescents’ closeness to their teachers had no unique effects on their subsequent school well-being and academic achievement after the transition. These results are in line with research that suggests that relational stressors are stronger risk factors than the absence of relational support mechanisms (Hamre and Pianta 2001; Spilt et al. 2012). One possible explanation for the relatively stronger results for post-transition conflict with teachers compared to the post-transition closeness to teachers is that adolescents may have only briefly known their seventh-grade teachers at the time of post-transition measurement. Due to the different subject teacher system of the seventh-grade environment (i.e., different teachers teaching different academic subjects), it is possible that close and supportive relationships with new teachers had not yet formed at the time of this study’s measurement. In contrast, if adolescents had faced major conflicts with their new teachers, these challenges would have been perhaps more visible immediately after the transition and had strong negative effects on the adolescents’ post-transition well-being and achievement.

Furthermore, the results supported the hypothesis that adolescents’ high school satisfaction would indirectly promote subsequent academic achievement through increased closeness to parents and teachers, whereas high school stress would indirectly hinder their subsequent achievement through increased conflicts with teachers. It is possible that low school well-being preceded more distant and strained relationships with parents and teachers (Kiuru et al. 2015; Pomerantz and Eaton 2001; Silinskas et al. 2015), for example, through the transmission of negative emotions while interacting with parents and teachers and through parents’ and teachers’ increased concern and their attempts to help and control the child. Thus, low quality relationships with parents and teachers would have further detrimental effects on adolescents’ academic achievement.

Finally, the analyses did not fully support one of the hypotheses: school well-being did not predict subsequent academic achievement through perceived quality of relationships with friends. One possible explanation for this lack of association is that school well-being may be partly shared in the peer group through emotional contagion and co-rumination of school-related affects and experiences (Kiuru et al. 2008; Lynch et al. 2013; Wang et al. 2018). It is possible that some of the mediating mechanisms occur at the peer group level. In future studies, it is important to investigate peer group phenomena related to school well-being and academic achievement, in addition to perceived closeness and conflict in relationships with school friends.

Overall, the results highlighted several mediating mechanisms that explain how the quality of interpersonal relationships and school well-being work together to predict subsequent achievement during educational transitions. Knowledge of the mechanisms that govern how interpersonal environments promote or hinder academic achievement is crucial for any applied research, involving prevention or intervention efforts for improving students’ academic achievement. These findings underscore the view that the promotion of students’ interpersonal relationships is an important tool in any interventions, aimed at helping adolescents deal with various academic challenges.

The researched phenomenon was also found to be a two-way street: high school well-being promoted higher quality interpersonal relationships, which in turn supported subsequent academic achievement. High levels of school-related stress had detrimental effects on academic achievement; however, these effects were indirect, instead operating through increased difficulties (i.e., conflicts) in interpersonal relationships. Such an accumulation of negative or positive school experiences during the transition to lower secondary school may have long-term consequences for an adolescent’s later school performance. The results suggest a need for theoretical models that allow describing more complex associations than merely focusing on the effects arising from parents’, friends’, and teachers’ relationships on later academic skills. According to the transactional theories (Sameroff 2009) underlying this study, both evocative and socialization effects should be considered. The results of this study suggest that it is important to support both adolescent school well-being and the quality of their interpersonal relationships when attempting to promote learning outcomes and reducing challenges related to educational transitions.

### Limitations and Future Directions

This study also has its limitations. First, the present study investigated only adolescents’ subjective well-being at school. In future studies, it would be important to investigate other aspects of school well-being, such as health status, school conditions (e.g., safety), or means for self-fulfillment in the school context (Konu and Lintonen 2005). Other important challenges for future studies would be to examine the roles of school experiences and school well-being in adolescents’ overall well-being (Markkanen et al. 2019; Salmela-Aro et al. 2009), as well as to examine school experiences at daily level. Also, possible spillover effects between different types of social relationships would be worthwhile to be studied (Kiuru et al. 2015). Second, despite the cross-lagged longitudinal design, where rank-order stabilities were statistically controlled, the analyses were nevertheless correlational, which inhibits confident assertions on causality. Third, although information about the adolescents’ academic achievement was retrieved from the school registers, the measurement of school well-being and perceived quality of interpersonal relationships were based on adolescents’ self-reports. In future studies, it would be important to use multiple reporters (e.g., parents, friends, and teachers) to triangulate data and investigate relationship quality also from the perspectives of the parents, friends, and teachers. There are some previous findings to suggest that perceptions of shared events can widely vary between different respondents (Cheung et al. 2016; Smetana 1995). Fourth, the present study was carried out in a particular cultural and educational environment (i.e., Finland) within a particular historical time. This may limit generalizability of the results to other contexts. It would be worthwhile to replicate the findings in other cultural and educational environments. Fifth, the present study only investigated the mechanisms mediating the effects of the quality of interpersonal relationships and school well-being on adolescents’ academic achievement. A challenge for future researchers would be to investigate multiple mediators and longer mediator chains, such as investigating possible motivation-related mediators as intervening mechanisms. Finally, the investigation of potential moderators (e.g., adolescent temperament or learning difficulties) among the associations between quality of interpersonal relationships and school well-being in regard to adolescents’ academic achievement remains a challenge for future research.

## Conclusion

Educational transitions allow researchers to explore how school well-being and interpersonal relationships relate to academic achievement over time. This study offered a novel insight into the dynamics between school well-being, the quality of interpersonal relationships, and academic achievement during the transition from primary to lower secondary school. Results suggest that adolescents’ high-quality interpersonal relationships with parents and peers that are characterized by closeness, encouragement, and support serve as developmental assets for negotiating school transitions. In contrast, conflicts with new teachers after the transition appeared to act as a risk factor. Thus, school policies and structures aimed at enhancing a lower secondary school teacher’s ability to connect with students in emotionally supportive ways may prevent the accumulation of non-supportive classroom experiences and other potential threats to learning outcomes. Results also highlighted several mediating mechanisms that elucidate how the quality of interpersonal relationships and school well-being work together to predict subsequent academic achievement during educational transitions. These findings underscore the importance of leveraging adolescents’ interpersonal relationships as an intervention tool to help them cope with various academic challenges.
